# Direct Inhibition of GSDMD by PEITC Reduces Hepatocyte Pyroptosis and Alleviates Acute Liver Injury in Mice

**DOI:** 10.3389/fimmu.2022.825428

**Published:** 2022-01-31

**Authors:** Jie Wang, Ke Shi, Ning An, Shuaifei Li, Mei Bai, Xudong Wu, Yan Shen, Ronghui Du, Jingcai Cheng, Xuefeng Wu, Qiang Xu

**Affiliations:** ^1^State Key Laboratory of Pharmaceutical Biotechnology, Nanjing Drum Tower Hospital, School of Life Science, Nanjing University, Nanjing, China; ^2^Drug R&D Institute, JC (Wuxi) Company, Inc., Wuxi, China

**Keywords:** PEITC, pyroptosis, hepatocyte, gasdermin D, acute liver injury

## Abstract

Acute liver injury (ALI), often caused by viruses, alcohol, drugs, etc., is one of the most common clinical liver diseases. Although pyroptosis plays an important role in ALI, there is still a lack of effective clinical drugs related to this mechanism. Here, we show that phenethyl isothiocyanate (PEITC), a natural compound present in cruciferous vegetables, can significantly alleviate concanavalin A (ConA)-induced inflammatory liver damage and carbon tetrachloride (CCl_4_)-induced chemical liver damage in a dose-dependent manner. PEITC dose-dependently reversed the ALI-induced increase in plasma levels of aspartate aminotransferase (AST), alanine aminotransferase (ALT), lactate dehydrogenase (LDH), tumor necrosis factor (TNF)-α, and interferon (IFN)-γ and reduced the protein levels of hepatocyte pyroptosis markers such as Nod-like receptor family pyrin domain containing 3 (NLRP3), cleaved caspase-1, and cleaved gasdermin D (GSDMD). *In vitro* experiments have also verified the inhibitory effect of PEITC on hepatocyte pyroptosis. Furthermore, PEITC inhibits pyroptosis by interacting with cysteine 191 of GSDMD. In summary, our findings establish a role for PEITC in rescuing hepatocyte pyroptosis *via* direct inhibition of GSDMD, which may provide a new potential therapeutic strategy for ALI.

## Introduction

Acute liver injury (ALI) is one of the most common clinical liver diseases and is often caused by viruses, alcohol, drugs, etc. If it is not treated in time, it can easily develop into acute liver failure, chronic liver injury, or hepatocellular carcinoma, which seriously threatens human quality of life and health. The mechanisms and characteristics of ALI are very complex, including hepatocyte death, oxidative stress, inflammation, etc. Hepatocyte pyroptosis is particularly critical for liver damage in the development of liver inflammation into cirrhosis, liver fibrosis, and hepatocellular carcinoma (1–4). In mouse sepsis models induced by lipopolysaccharide (LPS), the level of pyroptosis is positively correlated with the severity of liver injury (5). Signaling pathways involved in pyroptosis in hepatocytes and nonparenchymal cells were significantly enhanced in concanavalin A (ConA)-induced mouse ALI models (6).

Pyroptosis is a programmed cell death mediated by gasdermin family proteins (7), and its activation mechanism is divided into classical pathways and nonclassical pathways according to whether it depends on caspase-1 (CASP1). In the classical pathway, CASP1 is recruited and activated by inflammasomes. Activated CASP1 cleaves gasdermin D (GSDMD) to produce an active GSDMD-N-terminal domain. The GSDMD-N-terminal domain translocates to the cell membrane to form membrane pores, resulting in pyroptosis. In the nonclassical pathway, human caspase-4, caspase-5, and mouse caspase-11 can be directly activated by LPS and then cleave GSDMD, causing pyroptosis (8–10). In addition, there is a pyroptosis pathway that depends on caspase-3, which is an executive protein of apoptosis. When apoptotic cells are not cleared in time, the expression levels of gasdermin E (GSDME) are upregulated by p53 and then specifically cleaved by caspase-3 to produce the GSDME N-terminal domain (11). Finally, the GSDME-N-terminal domain forms membrane pores to induce pyroptosis (11, 12).

Although the liver has a regenerative function, ALI may develop into acute liver failure with extremely high mortality if liver regeneration cannot offset a large number of hepatocyte deaths (13). When acute liver injury lasts for more than 1 month, it will develop into chronic liver injury and liver cancer. At present, there is still a lack of effective clinical drugs to prevent and treat ALI. Therefore, exploring the occurrence and development mechanisms of ALI and finding safe and effective hepatoprotective drugs are urgent problems for researchers to solve.

Phenethyl isothiocyanate (PEITC) is an isothiocyanate compound from the secondary metabolites of cruciferous plants hydrolyzed by myrosinase (14). Isothiocyanates are a class of compounds containing RN=C=S, in which R can be aliphatic or aromatic and the group =C=S has high reactive affinity with cysteine (15). PEITC has antibacterial, anti-inflammatory, antioxidant, and antitumor effects (16–19). This compound is reported as an effective inducer of nuclear factor erythroid 2-related factor 2 (NRF2) and can promote the expression of a variety of NRF2-driven antioxidant genes in different cell types (20, 21). It also suppresses translocation of nuclear factor kappa B (NF-κB) and phosphorylation of its upstream inhibitor IκBα, thereby inhibiting the production of inflammatory factors (22). In addition, many other studies show that PEITC at high doses also has the ability to reduce cancer cell invasion and migration, block the cell cycle, and induce apoptosis and autophagy in cancer cells with increased production of ROS (23–25). Moreover, as a widely distributed natural compound, PEITC actually has an excellent protective effect on vital organs in the human body, although it may have been ignored thus far.

In this study, we discovered that PEITC has a significant hepatoprotective effect, which reverses the increased levels of aspartate aminotransferase (AST), alanine aminotransferase (ALT), lactate dehydrogenase (LDH), tumor necrosis factor (TNF)-α, and interferon (IFN)-γ in mice with ALI. Furthermore, this effect of PEITC may be due to inhibition of hepatocyte pyroptosis *via* its unique structure of mercapten-targeting cysteine 191 of GSDMD.

## Materials and Methods

### Chemicals and Reagents

PEITC was from JC (Wuxi) Company, Inc. (Wuxi, China). CCl_4_ and olive oil were purchased from Nanjing Chemical Reagent Co., Ltd. (Nanjing, China). Lactate dehydrogenase (LDH) assay kit, alanine aminotransferase (ALT) assay kit, and aspartate aminotransferase (AST) assay kit were from Nanjing Jiancheng Bioengineering Institute (Nanjing, China). A mouse TNF-α ELISA kit and IFN-γ ELISA kit were purchased from Dakewe Biotech Co., Ltd. (Shenzhen, China). Dulbecco’s modified Eagle’s medium/nutrient mixture F-12 (DMEM/F12), Lipofectamine 3000, and Alexa Fluor 488-conjugated anti-rabbit IgG were purchased from Thermo Fisher Scientific (Waltham, MA, USA). ExFect Transfection Reagent was purchased from Vazyme (Nanjing, China). Dexamethasone, adenosine triphosphate (ATP), penicillin–streptomycin solution (100×), cell lysis buffer for Western blotting and immunoprecipitation, and RIPA lysis buffer were provided by Beyotime Biotechnology (Shanghai, China). Insulin-Transferrin-Selenium (ITS) Media Supplement (100×) was purchased from R&D Systems (Minneapolis, MN, USA). LPS and ConA were bought from Sigma-Aldrich (St. Louis, MO, USA). Necrosulfonamide (NSA) was purchased from MedChemExpress (Monmouth Junction, NJ, USA). Antibodies against β-tubulin, β-actin, and GAPDH were purchased from Abmart (Shanghai, China). Antibody against Nod-like receptor family pyrin domain containing 3 (NLRP3) was purchased from Cell Signaling Technology (Danvers, MA, USA). Anti-caspase-1 and anti-IL-1β antibodies were provided by Santa Cruz Biotechnology (Santa Cruz, CA, USA). Anti-GSDMD antibody was from Abcam (Cambridge, UK).

### Animal Experiments

ICR male mice (6–8 weeks old) were purchased from the College of Veterinary Medicine Yangzhou University (Institute of Comparative Medicine, Yangzhou, China) and grown in an SPF-grade animal room at 21°C ± 2°C with a 12:12-h day-night cycle. PEITC (molecular mass: 163.24) is fat soluble, so it was dissolved in olive oil to the required concentrations.

#### ConA-Induced Acute Immune Liver Injury Model

Sixty ICR male mice were randomly divided into six groups: normal group, ConA-treated group, 7.5 mg/kg PEITC preadministered (pre) group, 15 mg/kg PEITC (pre) group, 30 mg/kg PEITC (pre) group, and 30 mg/kg PEITC postadministered (post) group. In the PEITC (pre) group, mice were given PEITC (7.5, 15, and 30 mg/kg, i.g.) for three consecutive days. After the last administration, the ConA-treated group, PEITC (pre) group, and PEITC (post) group were injected with 10 mg/kg ConA *via* the tail vein to induce an acute immune liver injury model. Half an hour after ConA injection, the PEITC (post) group was given PEITC (30 mg/kg, i.g.). Eight hours after ConA injection, blood was collected, and serum was separated by centrifugation. The mice were then sacrificed and rapidly dissected, and the livers and spleens of the mice were removed and weighed. In addition, liver and spleen index were calculated. The lesions on the surface of liver and spleen tissues were photographed and recorded. At the same time, a piece of liver tissue was removed. One part was fixed with paraformaldehyde, embedded in paraffin and sectioned, and the other part was stored at −80°C.

#### CCl_4_-Induced Acute Chemical Liver Injury Model

Male ICR mice were randomly divided into six groups: normal group, CCl_4_ model group, 7.5 mg/kg PEITC (pre) group, 15 mg/kg PEITC (pre) group, 30 mg/kg PEITC (pre) group, and 30 mg/kg PEITC (post) group. In the PEITC (pre) group, mice were given PEITC (7.5, 15, and 30 mg/kg, i.g.) for three consecutive days. After the last administration, the CCl_4_ model group, PEITC (pre) group and PEITC (post) group were injected with 0.2% (*v*/*v*) CCl_4_ (10 ml/kg, i.p.) to induce acute chemical liver injury. Half an hour and 8 h after CCl_4_ injection, the PEITC (post) group was given PEITC (30 mg/kg, i.g.). Sixteen hours after CCl_4_ injection, blood was collected, mice were euthanized, and liver tissue samples were removed.

### Analysis of Biochemical Indexes and Cytokine Levels

ALT, AST, and LDH activities were detected according to the instructions of the assay kits. The levels of serum TNF-α and IFN-γ were tested according to the instructions of ELISA kits.

### Real-Time PCR

Total RNA of liver tissues was extracted with TRIzol and then reverse transcribed into cDNA (26). Real-time quantitative PCR was performed with a BioRad CFX96 Touch™ real-time PCR detection system (BioRad, Hercules, CA, USA). The amplification conditions were 95°C for 2 min, 1 cycle, denaturation at 95°C for 10 s, annealing at 60°C for 30 s, extension at 95°C for 10 s, and 45 cycles. The primer sequences used in our study were as follows: TNF-α forward, 5′-CCT GTA GCC CAC GTC GTA G-3′; TNF-α reverse, 5′-GGG AGT AGA CAA GGT ACA ACC C-3′; IFN-γ forward, 5′-ATG AAC GCT ACA CAC TGC ATC-3′; IFN-γ reverse, 5′-CCA TCC TTT TGC CAG TTC CTC-3′; CXCL-10 forward, 5′-CCA AGT GCT GCC GTC ATT TTC-3′; CXCL-10 reverse, 5′-GGC TCG CAG GGA TGA TTT CAA-3′; β-actin forward, 5′-GGC TGT ATT CCC CTC CAT CG-3′; and β-actin reverse, 5′-CCA GTT GGT AAC AAT GCC ATG T-3′.

### Western Blot

Cells or liver tissues were lysed in lysis buffer with protease inhibitor cocktail for 30 min on ice, and the supernatants were collected after cell lysates were centrifuged at 12,000×*g* for 10 min. After the protein concentrations of each sample were measured, proteins were separated by sodium dodecyl sulfate–polyacrylamide gel electrophoresis (SDS-PAGE) and electrotransferred to polyvinylidene fluoride (PVDF) membranes (Millipore, Burlington, MA, USA). Different antibodies were incubated according to the molecular weight of target proteins for Western blotting.

### Immunohistochemistry

Paraffin-embedded liver tissue sections were deparaffinized, rehydrated, subjected to antigen retrieval, blocked, and then incubated overnight at 4°C with antibodies against NLRP3, IL-1β, caspase-1, and GSDMD. After washing tissue sections with PBST, slides were stained according to the instructions of the anti-mouse/rabbit universal immunohistochemical detection kit (Proteintech, Wuhan, China) and finally counterstained with hematoxylin.

### Cell Culture

The alpha mouse liver 12 (AML12) cell line and HEK293T cell line were obtained from the National Collection of Authenticated Cell Cultures (Shanghai, China). AML12 cells were cultured in DMEM/F12 medium supplemented with 10% fetal bovine serum (FBS), ITS media supplement (1×), 40 ng/ml dexamethasone, and penicillin–streptomycin solution (1×) under an atmosphere of 5% (*v*/*v*) CO_2_ at 37°C. To induce the hepatocyte pyroptosis model, AML12 cells were treated with 100 ng/ml LPS for 3 h, then PEITC (0.3, 1, and 3 µM) for 1 h, and finally 5 mM ATP for 24 h. The cells were collected for subsequent experiments. HEK293T cells were cultured in DMEM supplemented with 10% FBS and penicillin–streptomycin solution (1×) under an atmosphere of 5% (*v*/*v*) CO_2_ at 37°C.

### Immunofluorescence Assay

AML12 cells were fixed, permeabilized, blocked, and then incubated overnight at 4°C with anti-GSDMD antibody. After washing with PBS, cells were exposed to Alexa Fluor 488-conjugated anti-rabbit IgG, and finally, cell nuclei were stained with DAPI.

### Cell Transfection Assay

Mouse GSDMD WT-EGFP, human GSDMD WT-EGFP, and human GSDMD C191A-EGFP plasmids were purchased from General Biology (Anhui, China). The mouse GSDMD WT-EGFP plasmid was transfected into AML12 cells, and human GSDMD WT-EGFP or human GSDMD C191A-EGFP plasmid was transfected into HEK293T cells for subsequent experiments.

### Cellular Thermal Shift Assay

After AML12 cells were incubated with DMSO or PEITC (3 μM) for 2 h, the cells were collected, washed, and resuspended in PBS. The cell suspension was divided into 10 aliquots and heated at the indicated temperatures. Finally, the cell supernatant at each temperature was analyzed by western blot. HEK293T cells transfected with human GSDMD WT-EGFP or human GSDMD C191A-EGFP plasmid were treated with DMSO, PEITC (3 μM) or NSA (3 μM) for 2 h, and the subsequent operations were basically the same as above.

### Microscale Thermophoresis Assay

After the mouse GSDMD WT-EGFP plasmid was transiently transfected into AML12 cells for 24 h, the cells were lysed with lysis buffer, and the supernatant was collected after centrifugation. PEITC was gradually diluted from 500 μM and added to an aliquot of cell lysis. They were mixed and sucked into Monolith NT.115 capillary tubes (Nanotemper Technologies, München, Germany). Finally, samples were detected by Monolith NT.115 (Nanotemper Technologies), and data were analyzed by MO.Affinity Analysis. HEK293T cells were transiently transfected with human GSDMD WT-EGFP or GSDMD C191A-EGFP plasmid for 24 h and then lysed with lysis buffer. The supernatant was collected after centrifugation. PEITC or NSA was gradually diluted from 500 μM and added to an aliquot of cell lysis. After samples were mixed, the subsequent operations were the same as above.

### Electron Microscope

After the cell samples were fixed with prefixed electron microscopy fixative (Servicebio, Wuhan, China), they were sent to Wuhan Servicebio Co., Ltd. (Wuhan, China) to complete the electron microscope scan.

### Statistical Analysis

The results are displayed as the mean ± SEM, and each experiment included triplicate sets. GraphPad Prism 8 Software (San Diego, CA, USA) was used to conduct all statistical analyses. Data were statistically evaluated by Student’s *t*-test or one-way analysis of variance (ANOVA) followed by Bonferroni *post-hoc* test. *p* < 0.05 was considered significant.

## Results

### PEITC Ameliorated ConA-Induced Acute Immune Liver Injury in Mice

**Figure 1:**
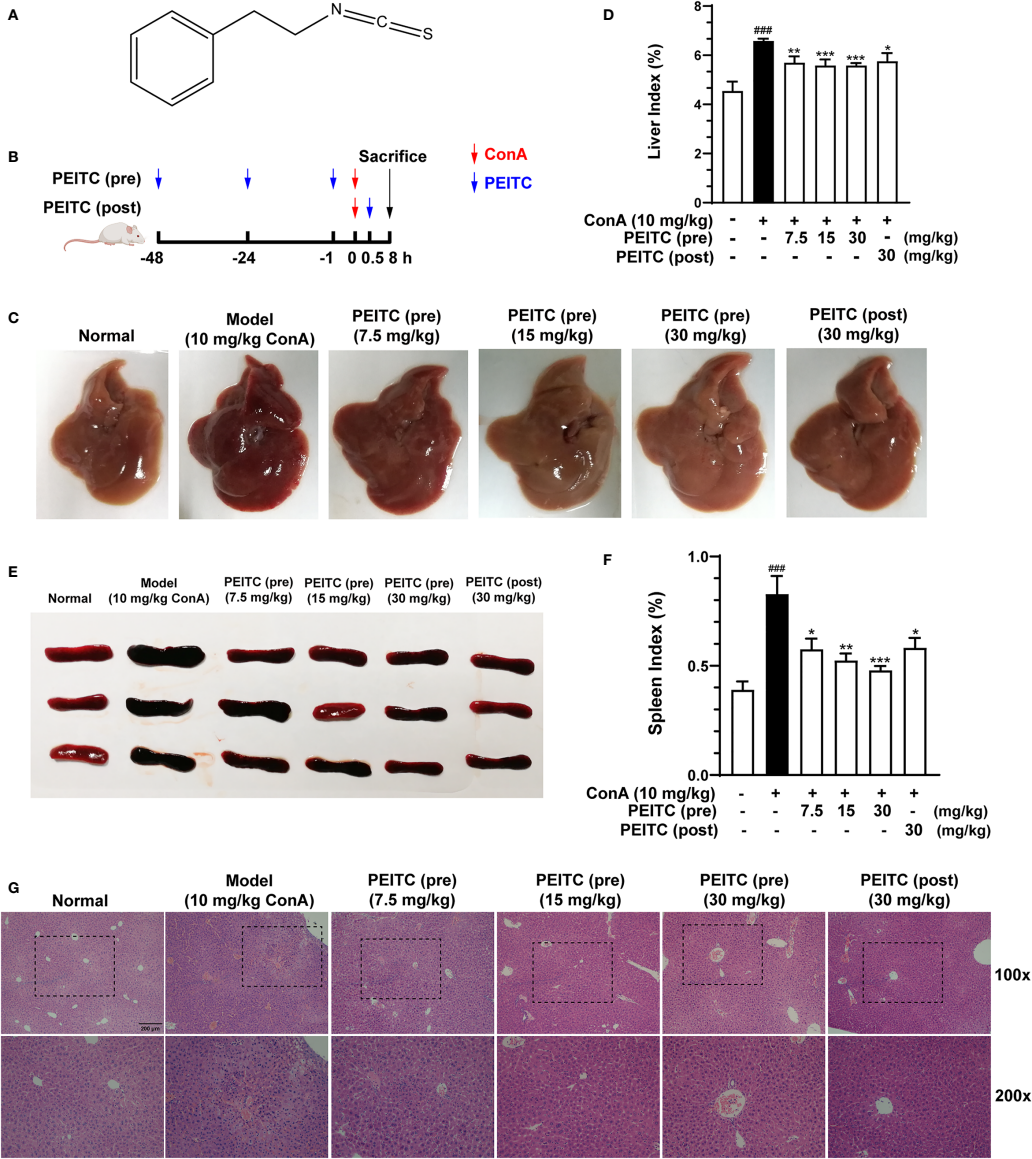
PEITC ameliorated the acute immune liver injury model induced by ConA in mice. **(A)** Chemical structure of PEITC. **(B)** Schematic overview of the ConA-induced acute immune liver injury model. **(C)** The lesions on the surface of liver tissues were photographed and recorded. **(D)** The liver indexes were measured. **(E)** The lesions on the surface of spleen tissues were photographed and recorded. **(F)** The spleen indexes were measured. **(G)** Hematoxylin and eosin (H&E) staining of liver tissue sections. Scale bar, 200 µm. Data are presented as means ± SEM (*n* = 10 per group). ^###^*p* < 0.001 vs. normal group, ^*^p < 0.05, ^**^p < 0.01, ^***^p < 0.001 vs. ConA control.

The molecular structure of PEITC is presented in **Figure 1A**. PEITC had no significant effect on the body weight of mice in the ConA-induced acute immune liver injury model (data not shown), indicating the safety and low toxicity of PEITC. According to the schematic diagram of this experiment (**Figure 1B**), after the mice were euthanized, the livers of mice from each group were removed and photographed. Compared with the normal group, the liver from the ConA-treated group was obviously congested and enlarged, and the liver lesions of mice in the PEITC (preadministered) and PEITC (postadministered) groups improved (**Figure 1C**). The livers of mice from each group were weighed, and the ratio of weight to body weight was calculated. We found that the liver index of mice in the ConA-treated group was significantly higher than that in the normal group, while the liver index of mice treated with PEITC decreased in a dose-dependent manner (**Figure 1D**). At the same time, the spleens of mice in the ConA-treated group were congested and swollen, and the spleen lesions of mice treated with PEITC were inhibited (**Figure 1E**). The spleen index of mice in the ConA-treated group was significantly increased in comparison with that of the normal group, while the spleen index of mice from the PEITC (pre) and PEITC (post) groups decreased in a dose-dependent manner, indicating that PEITC has a protective effect on the liver and spleen of mice with ConA-induced acute immune liver injury (**Figure 1F**). Hematoxylin and eosin (H&E) staining of liver tissue sections of mice from each group showed that compared with the normal group, a large number of erythrocytes were deposited in the liver sinusoids, the liver cords were disorderly arranged and the necrotic foci were diffusely distributed in the ConA-treated group. In the PEITC (pre) and PEITC (post) groups, the liver structure recovered in a dose-dependent manner, and sinusoidal congestion and inflammatory cell infiltration were significantly reduced (**Figure 1G**). The above results indicated that PEITC can obviously improve the liver and spleen tissue pathology of mice with ConA-induced acute immune liver injury.

### PEITC Reduced Hepatic Biochemical Indexes and Cytokine Levels

**Figure 2:**
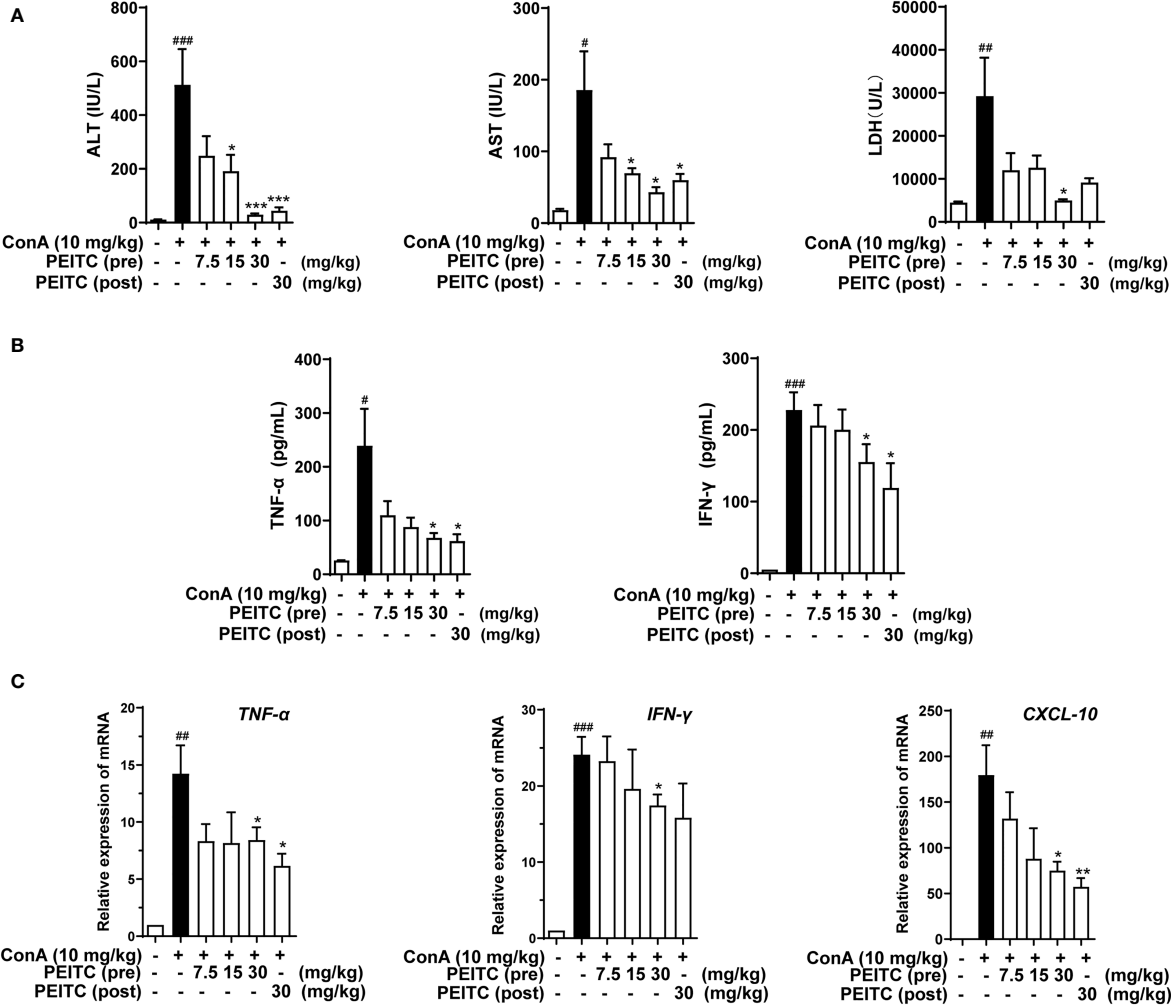
PEITC reduced hepatic biochemical indexes and cytokine levels. **(A)** The levels of serum ALT, AST, and LDH. **(B)** Serum TNF-α and IFN-γ levels. **(C)** The mRNA expression levels of TNF-α, IFN-γ, and CXCL-10 in liver tissues. Data are presented as means ± SEM (*n* = 10 per group). ^#^*p* < 0.05, ^##^*p* < 0.01, ^###^*p* < 0.001 vs. normal group, ^*^p < 0.05, ^**^p < 0.01, ^***^p < 0.001 vs. ConA control.

Next, we detected the levels of hepatic biochemical indexes ALT, AST, and LDH in the sera of mice from each group. The results showed that the levels of ALT, AST, and LDH in the ConA-treated group were significantly higher than those in the normal group, and PEITC inhibited the increase in ALT, AST, and LDH enzyme activities in the serum of ConA-treated mice in a dose-dependent manner (**Figure 2A**). ConA can activate macrophages, helper T cells, and natural killer T cells, which infiltrate the liver and produce a large number of proinflammatory cytokines, such as IFN-γ and TNF-α, as well as the chemokine CXCL-10. Here, we exploited ELISA to detect the effect of PEITC on the expression and secretion of inflammatory cytokines. PEITC significantly reversed the release of the inflammatory factors TNF-α and IFN-γ in the serum of ConA-treated mice (**Figure 2B**). In addition, PEITC also obviously downregulated the mRNA expression of TNF-α, IFN-γ, and CXCL-10 in the livers of mice with ALI (**Figure 2C**). These data indicated that PEITC can protect the liver and reduce cytokine levels in mice with ConA-induced acute immune liver injury.

### PEITC Prevented Hepatocyte Pyroptosis Induced by ConA

**Figure 3:**
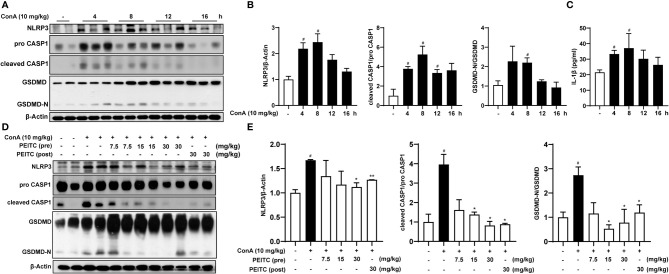
PEITC prevented hepatocyte pyroptosis induced by ConA. **(A)** Immunoblot analysis of NLRP3, pro-CASP1, cleaved CASP1, GSDMD, GSDMD-N, and β-actin in liver tissues of mice induced by ConA (0, 4, 8, 12, and 16 h). **(B)** The relative protein levels of NLRP3/β-actin, cleaved CASP1/pro-CASP1, and GSDMD-N/GSDMD are displayed as histograms. **(C)** The expression levels of serum IL-1β. **(D, E)** Western blot and statistical analysis of NLRP3, pro-CASP1, cleaved CASP1, GSDMD, GSDMD-N, and β-actin. Data are presented as the means ± SEM (*n* = 6–10 per group). ^#^*p* < 0.05 vs. normal group, ^*^p < 0.05, ^**^p < 0.01 vs. ConA control.

Since hepatocyte death is one of the important mechanisms of ALI, we further explored whether PEITC can directly protect hepatocytes from cell death. PEITC has an excellent anti-inflammatory effect, and pyroptosis, which is highly related to inflammation, plays a critical role in the process of ALI; therefore, we will focus on the effect of PEITC on hepatocyte pyroptosis. First, we verified the existence of hepatocyte pyroptosis in the ConA-induced ALI model. Western blotting was used to detect the expression levels of the pyroptosis-related proteins NLRP3, pro-CASP1, cleaved CASP1, GSDMD, and GSDMD-N in the livers of mice treated with ConA. We found that the expression of NLRP3 was upregulated, and the key proteins CASP1 and GSDMD were activated and cleaved in mice with ConA-induced ALI (**Figures 3A, B**). ELISA results also showed that the activity of serum IL-1β in ConA-treated mice was increased (**Figure 3C**). At the same time, these data suggested that hepatocyte pyroptosis was the most significant in mice when ConA was induced for 8 h. In the PEITC (pre) and PEITC (post) groups, the expression level of NLRP3 was downregulated, and the activation and cleavage of CASP1 and GSDMD were inhibited, indicating that PEITC can prevent ConA-induced ALI model mouse hepatocyte pyroptosis (**Figures 3D, E**).

### PEITC Reduced the Expression Levels of Pyroptosis-Related Proteins

**Figure 4:**
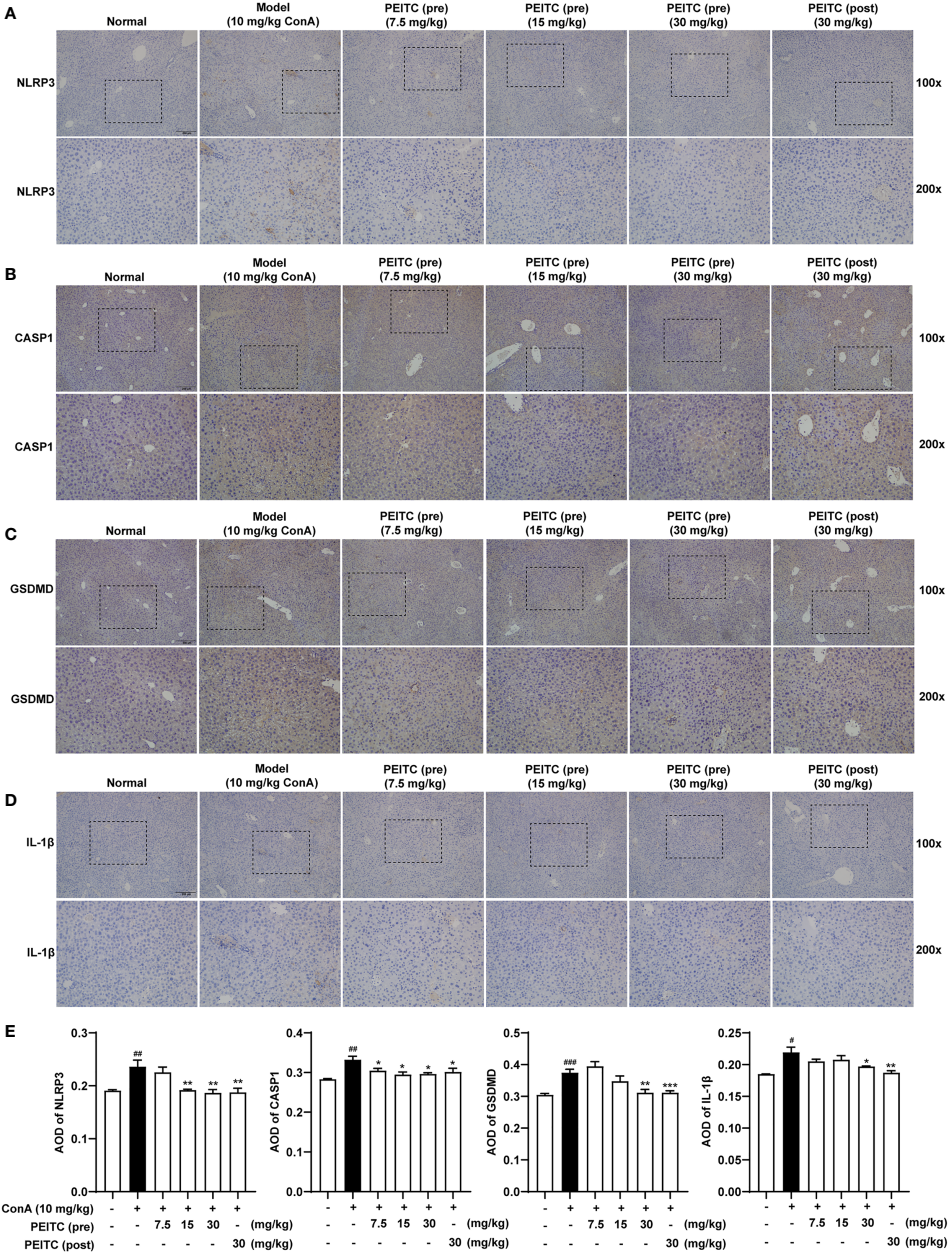
PEITC reduced the expression levels of pyroptosis-related proteins. **(A–D)** Liver tissue sections from each group were stained for NLRP3, CASP1, GSDMD, and IL-1β. Scale bar, 200 µm. **(E)** The average optical densities (AOD) of NLRP3, CASP1, GSDMD, and IL-1β are displayed as histograms. Data are presented as means ± SEM (*n* = 10 per group). ^#^*p* < 0.05, ^##^*p* < 0.01, ^###^*p* < 0.001 vs. normal group, ^*^p < 0.05, ^**^p < 0.01, ^***^p < 0.001 vs. ConA control.

Next, we performed immunohistochemical analysis on liver sections of mice from each group. The results of immunohistochemistry showed that the expression levels of NLRP3, IL-1β, CASP1 (pro- and cleaved CASP1) and GSDMD (GSDMD and GSDMD-N) increased in the ConA-treated group compared with the normal group (**Figures 4A–E**). The PEITC (pre) and PEITC (post) groups inhibited the expression levels of NLRP3, IL-1β, CASP1 (pro- and cleaved CASP1), and GSDMD (GSDMD and GSDMD-N) in a dose-dependent manner. These results confirmed that PEITC can reduce the expression of pyroptosis-related proteins in ConA-treated mice.

### PEITC Ameliorated Acute Chemical Liver Injury Model and Hepatocyte Pyroptosis Induced by CCl_4_ in Mice

**Figure 5:**
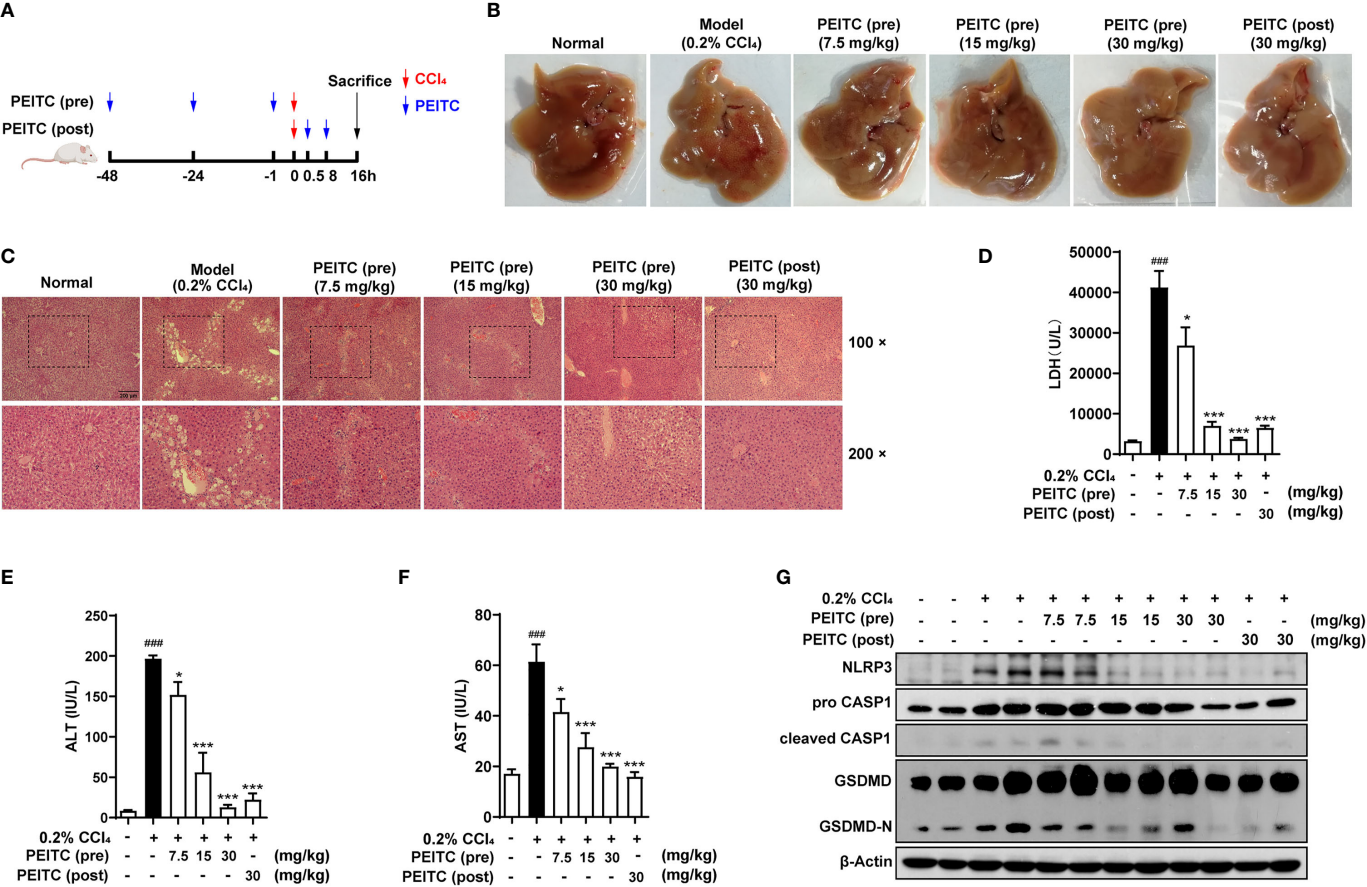
PEITC ameliorated the acute chemical liver injury model and hepatocyte pyroptosis induced by CCl_4_ in mice. **(A)** Schematic overview of the CCl_4_-induced acute chemical liver injury model. **(B)** The lesions on the surface of liver tissues were photographed. **(C)** H&E staining of liver tissue sections. Scale bar, 200 µm. **(D–F)** The levels of serum LDH, ALT, and AST. **(G)** Western blot analysis of NLRP3, pro-CASP1, cleaved CASP1, GSDMD, GSDMD-N, and β-actin. Data are presented as the means ± SEM (*n* = 6–10 per group). ^###^*p* < 0.001 vs. normal group, ^*^p < 0.05, ^***^p < 0.001 vs. CCl_4_ control.

To verify the general inhibitory effect of PEITC on hepatocyte pyroptosis in different ALI models, we conducted experiments in CCl_4_-induced acute chemical liver injury. The experimental process is shown in **Figure 5A**. We found that PEITC had no significant effect on the body weight of mice (data not shown). Compared with the normal group, the livers of mice in the CCl_4_ model group were yellowish, with a large number of granular dots on the surface (**Figure 5B**). PEITC restored the normal color and texture of mouse liver to varying degrees in the PEITC (pre) and PEITC (post) groups. H&E staining also indicated that the livers of mice in the CCl_4_ model group showed obvious damage, including hepatocyte necrosis, vacuolar degeneration, loss of hepatic cord structure, and a large amount of immune cell infiltration in the portal vein system (**Figure 5C**). Liver injury in mice from the PEITC (pre) and PEITC (post) groups showed a dose-dependent improvement. In addition, the levels of LDH, ALT and AST in the model group were also significantly higher than those in the normal group. The serum LDH, ALT and AST levels of mice in the PEITC (pre) and PEITC (post) groups were obviously reversed in a dose-dependent manner (**Figures 5D–F**). At the same time, we detected the expression levels of pyroptosis-related proteins in the liver of mice from each group and found that the liver of mice from the model group had significant hepatocyte pyroptosis, and the expression of pyroptosis-related proteins in the liver tissue of the PEITC (pre) and PEITC (post) groups was downregulated (**Figure 5G**). These data determined that PEITC can also improve CCl_4_-induced acute chemical liver injury by inhibiting hepatocyte pyroptosis.

### PEITC Inhibited AML12 Pyroptosis Induced by LPS and ATP *In Vitro*

**Figure 6:**
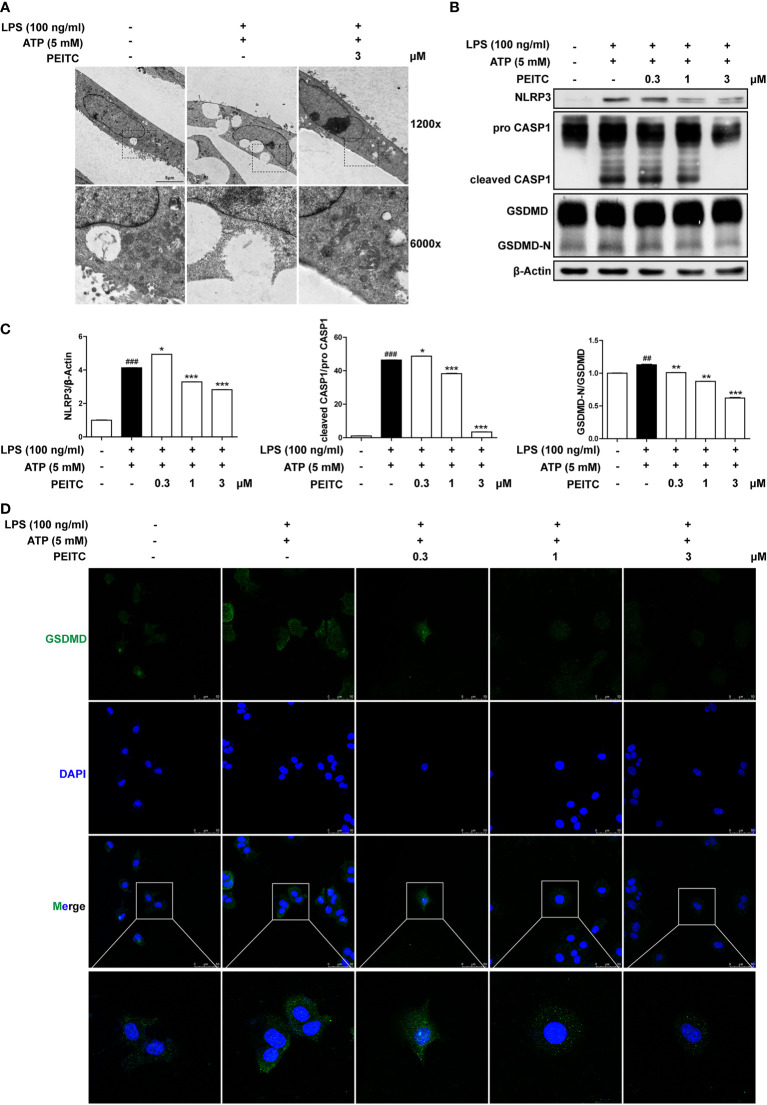
PEITC inhibited pyroptosis in AML12 cells treated with LPS and ATP *in vitro*. **(A)** Representative electron micrographs of cell morphology from each group. Scale bar, 5 µm. **(B, C)** Immunoblot and statistical analysis of NLRP3, pro-CASP1, cleaved CASP1, GSDMD, GSDMD-N, and β-actin. **(D)** Immunofluorescence staining of GSDMD. Scale bar, 50 µm. Data are presented as means ± SEM. ^##^*p* < 0.01, ^###^*p* < 0.001 vs. blank, ^*^p < 0.05, **p < 0.01 ^***^p < 0.001 vs. LPS+ATP control.

We further verified the inhibitory role of PEITC on hepatocyte pyroptosis *in vitro*. Because pyroptosis has obvious morphological characteristics, we first observed the effect of PEITC on the morphology of pyroptotic cells through electron microscopy. The typical characteristics of pyroptosis in AML12 cells induced by LPS plus ATP include cell swelling, rupture of the cytoplasmic membrane, and intracellular vacuoles, while the morphology of AML12 cells treated with PEITC was restored, the plasma membrane was more complete, and intracellular vacuoles were reduced (**Figure 6A**). Immunoblot assays also showed that PEITC inhibited the increase in NLRP3 expression and the activation and cleavage of CASP1 and GSDMD in AML12 cells induced by LPS and ATP (**Figures 6B, C**). Furthermore, immunofluorescence results indicated that the expression level of GSDMD (GSDMD and GSDMD-N) increased and concentrated on the plasma membrane in AML12 cells induced by LPS plus ATP, while PEITC treatment reduced the GSDMD level in a dose-dependent manner (**Figure 6D**). These results verified the function of PEITC in inhibiting hepatocyte pyroptosis *in vitro*.

### PEITC Directly Inhibited GSDMD by Interacting With Cysteine 191 of GSDMD

**Figure 7:**
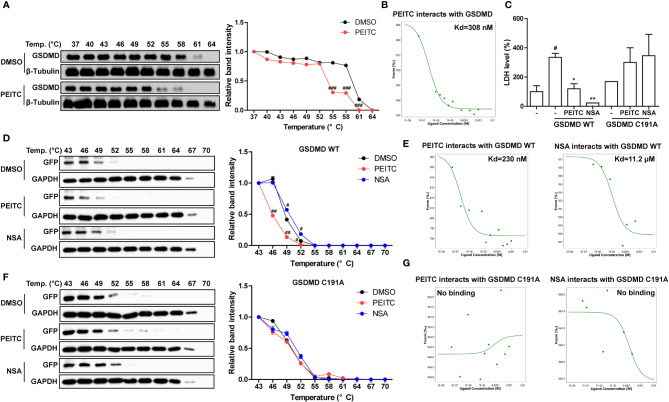
PEITC directly inhibited GSDMD by interacting with cysteine 191 of GSDMD. **(A)** The effect of PEITC on the thermal stability of mouse GSDMD was detected by CETSA, and the line graph was used to show the remaining GSDMD amount at different temperatures. **(B)** The binding ability between PEITC and mouse GSDMD was detected by MST. **(C)** The secretion levels of LDH in cell culture supernatants. **(D)** The effect of PEITC and NSA on the thermal stability of human GSDMD WT. **(E)** The binding ability between human GSDMD WT and PEITC or NSA. **(F)** The effect of PEITC and NSA on the thermal stability of human GSDMD C191A. **(G)** The binding ability between human GSDMD C191A and PEITC or NSA. Data are presented as means ± SEM. ^#^*p* < 0.05, ^##^*p* < 0.01, ^###^*p* < 0.001 vs. DMSO or blank, ^*^p < 0.05, ^**^p < 0.01 vs. GSDMD WT control.

Since PEITC has a significant inhibitory effect on the cleavage of the pore-forming effector protein GSDMD during pyroptosis *in vivo* and *in vitro*, we tried to detect whether PEITC can directly interact with GSDMD. Molecular docking analysis predicted that PEITC binds to human GSDMD (PDB ID: 6N9O, -CDOCKER INTERACTION ENERGY = 28.859, data not shown). The cellular thermal shift assay (CETSA) showed that mouse GSDMD was completely degraded at 64°C in the DMSO group, while PEITC treatment accelerated the degradation of mouse GSDMD at 61°C and reduced the thermal stability of mouse GSDMD (**Figure 7A**). The MST results indicated that the Kd value between PEITC and mouse GSDMD was 308 nM (**Figure 7B**). PEITC has high reactive affinity for cysteine, and cysteine 191/192 of human/mouse GSDMD is essential to its membrane pore formation function. Therefore, we constructed a human GSDMD WT-EGFP plasmid and GSDMD C191A-EGFP plasmid for subsequent experiments and used NSA (inhibitor of GSDMD) as a positive control. By detecting the content of LDH in the medium supernatant, we found that the amount of LDH released from HEK293T cells transfected with C191A-GSDMD was less than that released from cells transfected with WT-GSDMD. PEITC and NSA inhibited the amount of LDH released from HEK293T cells transfected with WT-GSDMD (**Figure 7C**). The CETSA results showed that PEITC reduced the thermal stability of human WT-GSDMD, while NSA enhanced the thermal stability of human WT-GSDMD (**Figure 7D**). The MST results indicated that the Kd value between PEITC and human WT-GSDMD was 230 nM, and the Kd value between NSA and human WT-GSDMD was 11.2 μM (**Figure 7E**). However, PEITC and NSA had no significant effect on the thermal stability of human C191A-GSDMD, and PEITC and NSA did not bind to human C191A-GSDMD (**Figures 7F, G**). The above results confirmed that PEITC can directly bind to human GSDMD through cysteine 191 of GSDMD.

## Discussion

ALI is one of the most common clinical liver diseases with complicated pathogenesis. Since the liver is responsible for the material metabolism and detoxification of the body, it is very susceptible to damage (27). Parenchymal hepatocytes, Kupffer cells, lymphocytes, and hepatic stellate cells all play a role in the occurrence and development of ALI. The key features of ALI include cell death, inflammation, and oxidative stress (28, 29). Among them, hepatocyte pyroptosis may be particularly critical (6). However, there are very few drugs clinically used to protect the liver and to treat ALI. If ALI is not treated in time, it can easily develop into acute liver failure, chronic liver injury, or hepatocellular carcinoma, which seriously threatens human health and quality of life. Therefore, it is necessary to find clinical prevention and treatment drugs for ALI with fewer side effects and better curative effects.

PEITC has been reported to be used as an antibacterial, anti-inflammatory, antioxidant and anticancer agent (22, 30, 31). In a clinical trial about PEITC in the treatment of prostate hyperplasia, we found that many elderly patients had decreased liver function and increased serum transaminase levels, while the serum transaminase levels of elderly patients treated with PEITC significantly decreased. This suggests that PEITC may have a hepatoprotective function.

Here, we exploited a ConA-induced acute immune liver injury model and a CCl_4_-induced acute chemical liver injury model to explore the hepatoprotective effect of PEITC (**Figures 1B**, **5A**). ConA, a kind of T-cell mitogen, can induce ALI in mice by activating T lymphocytes. ALI induced by CCl_4_ can mimic the mechanism of ALI caused by chemical drugs. CCl_4_ can cause the peroxidation of cell membrane lipids and proteins and destroy calcium ion channels to increase intracellular calcium ion concentrations, which will eventually lead to the death of liver cells and cause intracellular inflammation. PEITC significantly improved the ConA-induced acute immune liver injury model and CCl_4_-induced acute chemical liver injury model. Compared with the model group, the normal structural units and material metabolism function of the liver in the PEITC (pre) group and PEITC (post) group were restored in a dose-dependent manner, and the activity of serum ALI-related biochemical indicators ALT, AST, and LDH was downregulated (**Figures 1**, **2A**, **5**). In addition, the expression and release of inflammatory factors were reduced in the livers of mice from the PEITC (pre) group and PEITC (post) group (**Figures 2B, C**, **4D**). It should be noted that pretreatment with PEITC (30 mg/kg) tended to have stronger effects on its inhibitory properties than posttreatment. PEITC is the most widely studied aromatic isothiocyanate with high bioavailability. This compound has rapid absorption, a low clearance rate and a high protein binding rate after oral administration, which may be due to its lipophilicity and low molecular weight (15). It has been reported that the peak time of plasma concentration is 2.6 ± 1.1 h and the half-life period is 4.9 ± 1.1 h after PEITC is orally taken (32). In our ALI experiment, PEITC was preadministered once a day for three consecutive days, while it was postadministered only once after ConA injection. Therefore, the blood drug concentrations in the PEITC (pre) group might be much higher than those in the PEITC (post) group when the livers were injured with ConA.

In a previous report, it was shown that in ConA-treated mice, pathogenic-elevated NLRP3, cleaved caspase-1 and IL-1β levels, as well as inflammatory cell death known as pyroptosis, occurred in the liver (6). Here, we verified for the first time that pyroptosis, with cleaved GSDMD and pore formation in the membrane as the gold standard, actually occurs during inflammatory ALI caused by ConA (**Figures 3**, **4C**, **6**). Moreover, in this study, we found that hepatocytes also experience notable pyroptosis in the process of CCl_4_-induced chemical liver injury (**Figure 5**). Pyroptosis is a kind of programmed cell death that is characterized by the continuous expansion of cells until the rupture of the cell membrane. Under the electron microscope, it can be clearly seen that the cells underlying pyroptosis form a large number of vesicles. After that, pores will be formed on the cell membrane, the cell membrane will rupture, and the content will flow out, thus resulting in robust inflammation (33). These biochemical characteristics of pyroptosis are consistent with the phenomena revealed in this study. Furthermore, we found that PEITC reduced the production of NLRP3 and the cleavage of CASP1 and GSDMD in the livers of mice with ALI (**Figures 3D, E**, **4**, **5G**) and in AML12 cells *in vitro* (**Figure 6**). These results indicate that PEITC can significantly inhibit pyroptosis in hepatocytes.

Previous research shows that scavenging ROS by *N*-acetyl-cysteine is an effective strategy to attenuate NLRP3 inflammasome activation and to suppress liver inflammation (6). PEITC has also been reported to significantly increase the activities of Nrf2 and a series of antioxidant enzymes in fibroblasts (21). Mechanistic studies showed that the anti-inflammatory effect of PEITC was driven, at least in part, by inhibiting the NLRP3 inflammasome pathway, as indicated by the downregulation of NLRP3, TXNIP, caspase-1, and IL-1β (34, 35). Our results also showed that PEITC can inhibit the expression of NLRP3 and the cleavage of CASP1 (upstream of GSDMD) both *in vivo* and *in vitro* (**Figures 3**, **4A, B**, **5G**, **6B, C**). We speculate that this may be partly due to its anti-inflammatory effects and partly resulted from feedback regulation in pyroptosis inhibition. Although we cannot rule out other sites of action and other mechanisms of PEITC that might indirectly lead to its suppressive effects on pyroptosis, the efficacy of PEITC may be mainly due to its direct inhibition of GSDMD in hepatocytes (**Figures 7A, B**). Cysteine 191/192 in human/mouse GSDMD is highly conserved and essential for the formation of its oligomerization and pyroptosis pores (36, 37). Since PEITC containing –N=C=S has high-reactive affinity with cysteine, we subsequently explored whether cysteine 191 of human GSDMD affects the function of PEITC and its binding to GSDMD. By detecting the content of LDH in the medium supernatant, we found that PEITC treatment reduced the release of LDH from HEK293T cells transfected with the GSDMD WT plasmid but had no significant effect on the release of LDH from cells transfected with GSDMD C191A (**Figure 7C**). In addition, compared with HEK293T cells with WT GSDMD, the release of LDH from cells with C191A GSDMD was reduced, which verified the critical role of cysteine 191 in GSDMD pyroptosis. The CETSA and MST assays showed that PEITC could bind to human WT-GSDMD but not C191A-GSDMD (**Figures 7D**–**G**). These results determined that PEITC can directly interact with cysteine 191 of GSDMD to inhibit hepatocyte pyroptosis.

**Figure 8:**
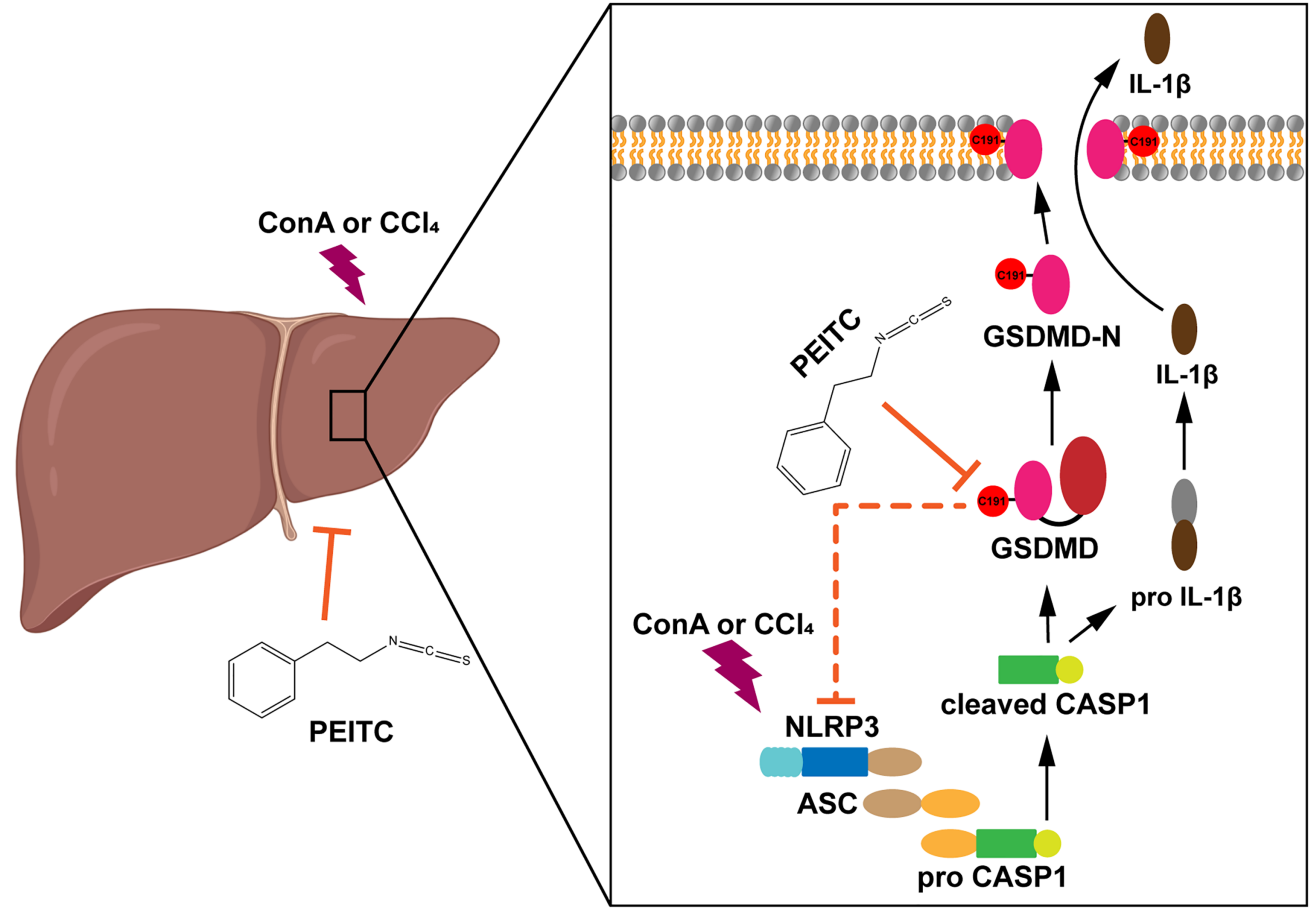
Diagram of the effect of PEITC in inhibiting hepatocyte pyroptosis.

In summary, our study reported for the first time the role of PEITC in inhibiting hepatocyte pyroptosis in ALI. We also found a new target of PEITC, GSDMD, and clarified the importance of cysteine 191 of GSDMD in the interaction between PEITC and GSDMD (**Figure 8**). These findings provide a new drug candidate for the clinical prevention and treatment of ALI.

## Data Availability Statement

The original contributions presented in the study are included in the article/**Supplementary Material**. Further inquiries can be directed to the corresponding authors.

## Ethics Statement

The animal study was reviewed and approved by the Nanjing University Animal Care and Use Committee (NJU-ACUC).

## Author Contributions

JW, KS, NA, SL, and MB performed research and analyzed the data. JW, KS, and XFW designed the experiments and wrote the paper. XDW, YS, and RD contributed experimental materials and gave suggestions in the formation. JC, XFW, and QX designed the research and obtained funding. All authors listed have made a substantial, direct, and intellectual contribution to the work and approved it for publication.

## Funding

This work was supported by the National Key R&D Program of China (No. 2017YFA0506000), National Natural Science Foundation of China (Nos. 81773798, 82073910, 21937005), Natural Science Foundation of Jiangsu Province (BK20191253), and Wuxi Science and Technology Development Fund (CBE01G1650).

## Conflict of Interest

Author JC was employed by company JC (Wuxi) COMPANY, Inc.

The remaining authors declare that the research was conducted in the absence of any commercial or financial relationships that could be construed as a potential conflict of interest.

## Publisher’s Note

All claims expressed in this article are solely those of the authors and do not necessarily represent those of their affiliated organizations, or those of the publisher, the editors and the reviewers. Any product that may be evaluated in this article, or claim that may be made by its manufacturer, is not guaranteed or endorsed by the publisher.
