# Supplementary material for: Direct Inhibition of GSDMD by PEITC Reduces Hepatocyte Pyroptosis and Alleviates Acute Liver Injury in Mice
Source: Front Immunol. 2022 Jan 31;13:825428. doi: 10.3389/fimmu.2022.825428 (PMC8841757; doi:10.3389/fimmu.2022.825428)
Supplement: Supplementary file 1 [file Presentation_1.zip › supplementary materials/Figure 3/Figure 3A (Representative WB).docx]

Pro CASP1:





Cleaved CASP1:





GSDMD GSDMD-N:
