# Supplementary material for: Direct Inhibition of GSDMD by PEITC Reduces Hepatocyte Pyroptosis and Alleviates Acute Liver Injury in Mice
Source: Front Immunol. 2022 Jan 31;13:825428. doi: 10.3389/fimmu.2022.825428 (PMC8841757; doi:10.3389/fimmu.2022.825428)
Supplement: Supplementary file 1 [file Presentation_1.zip › supplementary materials/Figure 7/Figures 7D, F/Figures 7D, F (Representative WB).docx]

GFP (GSDMD C191A NSA)

GAPDH (GSDMD C191A NSA)

GFP (GSDMD C191A PEITC)

GAPDH (GSDMD C191A PEITC)

GFP (GSDMD C191A DMSO)

GAPDH (GSDMD C191A DMSO)





High exposure:

GFP (GSDMD C191A NSA)

GAPDH (GSDMD C191A NSA)

GFP (GSDMD C191A PEITC)

GAPDH (GSDMD C191A PEITC)

GFP (GSDMD C191A DMSO)

GAPDH (GSDMD C191A DMSO)

GFP (GSDMD WT NSA)

GAPDH (GSDMD WT NSA)

GAPDH (GSDMD WT PEITC)





GFP (GSDMD WT PEITC)





GFP (GSDMD WT DMSO)





GAPDH (GSDMD WT DMSO)
